# Functional block copolymer micelles based on poly (jasmine lactone) for improving the loading efficiency of weakly basic drugs†

**DOI:** 10.1039/d2ra03962a

**Published:** 2022-09-21

**Authors:** Aliaa Ali, Rajendra Bhadane, Afshin Ansari Asl, Carl-Eric Wilén, Outi Salo-Ahen, Jessica M. Rosenholm, Kuldeep K. Bansal

**Affiliations:** Pharmaceutical Sciences Laboratory, Faculty of Science and Engineering, Åbo Akademi University, BioCity (3rd floor) Tykistökatu 6A 20520 Turku Finland jessica.rosenholm@abo.fi; Structural Bioinformatics Laboratory, Faculty of Science and Engineering, Biochemistry, Åbo Akademi University 20520 Turku Finland; Laboratory of Molecular Science and Engineering, Åbo Akademi University, Aurum Henrikinkatu 2 20500 Turku Finland kuldeep.bansal@abo.fi

## Abstract

Functionalization of polymers is an attractive approach to introduce specific molecular forces that can enhance drug–polymer interaction to achieve higher drug loading when used as drug delivery systems. The novel amphiphilic block copolymer of methoxy poly(ethylene glycol) and poly(jasmine lactone) *i.e.*, mPEG-*b*-PJL, derived from renewable jasmine lactone provides free allyl groups on the backbone thus, allowing flexible and facile post-synthesis functionalization. In this study, mPEG-*b*-PJL and its carboxyl functionalized polymer mPEG-*b*-PJL-COOH were utilised to explore the effect of ionic interactions on the drug–polymer behaviour. Various drugs with different p*K*_a_ values were employed to prepare drug-loaded polymeric micelles (PMs) of mPEG-*b*-PJL, mPEG-*b*-PJL-COOH and Soluplus® (polyvinyl caprolactam–polyvinyl acetate–polyethylene glycol graft copolymer) *via* a nanoprecipitation method. Electrostatic interactions between the COOH pendant on mPEG-*b*-PJL-COOH and the basic drugs were shown to influence the entrapment efficiency. Additionally, molecular dynamics (MD) simulations were employed to understand the polymer–drug interactions at the molecular level and how polymer functionalization influenced these interactions. The release kinetics of the anti-cancer drug sunitinib from mPEG-*b*-PJL and mPEG-*b*-PJL-COOH was assessed, and it demonstrated a sustainable drug release pattern, which depended on both pH and temperature. Furthermore, the cytotoxicity of sunitinib-loaded micelles on cancer cells was evaluated. The drug-loaded micelles exhibited dose-dependent toxicity. Also, haemolysis capacity of these polymers was investigated. In summary, polymer functionalization seems a promising approach to overcome challenges that hinder the application of polymer-based drug delivery systems such as low drug loading degree.

## Abstract 2

Block copolymer micelles with a functional core have been synthesized and evaluated for their drug delivery capability. High drug loading was observed due to strong ionic interactions, while cytotoxicity of polymers was found to be low.

## Graphical abstract

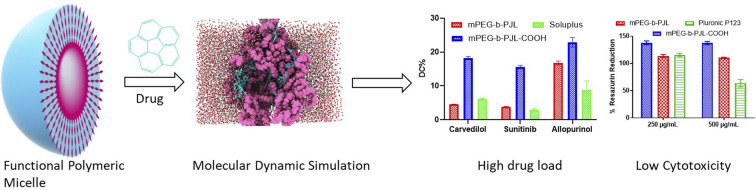

## Introduction

Polymeric micelles (PMs) are considered a promising carrier as they exhibit various features such as easier preparation methods, good loading capacity, and better formulation stability that prompt their usage in drug delivery applications.^1,2^ PMs generally feature a narrow size distribution in which the diameters of PMs are typically in the range of 10–100 nm.^3^ PMs are generated with amphiphilic polymers where the self-assembly of these molecules occurs at certain concentrations that are defined as the critical micellar concentration (CMC).^4^ Amphiphilic polymers synthesised from renewable feedstock is an interesting new aspect as they are considered a sustainable and a greener alternative to the fossil-based materials.^5^ The applications of PMs are correlated with their unique core–shell structure that allows encapsulation of hydrophobic drugs, proteins, or DNA *via* different physical or chemical binding modes.^6^ Hydrophobic drugs migrate towards the hydrophobic block (core) due to the hydrophobic interactions, forming drug-loaded micelles, while the hydrophilic block (shell) provides the steric stability to micelles as it maintains micelles well dispersed in aqueous solution without aggregation.^1^

In drug discovery, most hits derived from high-throughput screening (HTS), lead compounds, candidates under development as well as several marketed drugs are highly hydrophobic in nature, and it is challenging to enhance the water solubility of those drug candidates without compromising their potency.^7^ Hence, the ability of PMs to solubilize hydrophobic drugs within their core are greatly advantageous in drug development. In addition, they protect drug molecules from being inactivated in the biological milieu, which enhances bioavailability.^8^ For instance, poly(decalactone) micelles demonstrated their efficacy in enhancing the water solubility and stability of curcumin in physiologically relevant medium.^9^ However, despite the potential of PMs in drug delivery, they show several drawbacks that create a gap between their current extensive study status and clinical success. PMs suffer from thermodynamic instability because dilution below the CMC leads to destabilisation of micelle structure and causes premature drug release.^10^ For instance, *in vivo* studies showed that 74% of poly(ethylene glycol)-*b*-polycaprolactone (PCL) micelles with CMC of 38 mg L^−1^ were still found after 24 h of circulation in mouse blood compared to only 33% of Pluronic P85 (poly(ethylene oxide)-*b*-poly(propylene oxide)-*b*-poly(ethylene oxide)) micelles with higher CMC value, *i.e.*, 300 mg L^−1^.^11–13^ Moreover, PMs usually exhibit low drug loading, where drug loading degrees in most reported micellar systems are lower than 5% w/w.^14^ One approach to increase the amount of drug loading in micelle-based formulations is to increase the copolymer concentration. Nonetheless, the increase in copolymer concentration beyond a certain limit could also compromise the stability of the formulation as micelles tend to form aggregates as a result of the increased interactions. Besides, the solubility of the copolymer limits its concentration in a micellar solution.^15^ Furthermore, the large amounts of the polymeric carrier materials that are needed to administer required dose of the drug must be degraded and/or metabolised and excreted by the patient without showing any toxicity, which inflicts an extra burden on the patients.^16^ In general, the structure, as well as physical and chemical properties of the polymeric carrier determine the drug loading content.^17^ Furthermore, drug–carrier interactions influence drug release profiles, whereby drug molecules may directly interact with drug carriers thus retarding their release.^18^ Achieving high drug loading in most PMs still constitutes a major challenge. Besides, the stability of the formulation and the unsatisfactory drug release hinders the application of PMs.^19^

Hydrophobic interaction between the poorly aqueous soluble drug and the polymer is considered the main driving force for drug encapsulation. Nevertheless, being non-specific, hydrophobic interactions can also take place between free drug molecules causing drug aggregation during the self-assembly, which results in a decreased drug loading efficiency.^19^ It has been observed that non-covalent interactions such as hydrogen bonding, van der Waals (vdW) and electrostatic interactions can enhance drug–polymer interactions and are proven to offer higher drug loading capacity.^20^ Molecular dynamics (MD) simulations have successfully been employed for such intermolecular interaction studies.^21–24^

Giacomelli *et al.* encapsulated probes with weak carboxylic acid groups within micelles containing poly[2-(dialkylamino)ethyl methacrylate] cores that show weak basic properties. The probe/micelle systems that can form R1-COOH/NH2-R2 acid–base pairs within the micellar core exhibited high loading capacities that decreased remarkably with the esterification of the carboxylic acid moieties.^25^ Henceforth, the introduction of specific molecular forces capable of enhancing drug–polymer interaction could be the most promising approach to achieve higher drug loading.^19^ For example, Lee *et al.* fabricated PEO–PLA diblock copolymer containing carboxylic group *via* the catalytic debenzylation of its precursor formed by the ring-opening copolymerization of 3-(*s*)-[(benzyloxycarbonyl)methyl]-1,4-dioxane2,5-dione (BMD), methoxy terminated poly(ethylene oxide) (MePEO) and lactide (LAC). The weight percent of BMD carrying the COOH group ranged from 0 to 19.5% resulting in a varying amount of COOH. They found that the loading capacity of the investigated drug, papaverine, increased from 3.5 to 15% (wt/wt) as the COOH content increased.^26^ Similarly, Yang *et al.* synthesized a novel series of poly(carbonate) and poly(ethylene glycol) (PEG) block copolymers functionalized with urea and acid. The doxorubicin (DOX) micelles synthesized with this new functionalized polymer provided a high drug-loading capacity because of the strong ionic interaction between the acid in the polymer and the amine in DOX.^20^ Hedrick *et al.* have also shown that the functionalization of micelles with carboxylic acid groups increases the loading of DOX.^27^ Another work done by Zhu *et al.* showed that micelles based on Pluronic F127 could load a higher amount of bufalin with the use of COOH functional groups.^28^ Lv *et al.* took a slightly different approach through the incorporation of coordination interactions between electron acceptor-containing polymers and electron donor-containing drugs. They synthesized an amphiphilic copolymer containing phenylboronic acid (PBA) pendant to act as electron acceptor. The PBA pendant formed donor–acceptor coordination with doxorubicin that bears a primary amine group that acts as an electron donor. The resulting micelles achieved ultrahigh drug loading (∼50%).^19^

One of the simpler methods to generate polymer with tunable free functional groups is the post modification of “allyl” terminated polymers.^29^ However, existence of such polymers with free functional groups are scarce in literature. In our previous work, we have successfully synthesised block copolymers of poly(jasmine lactone) (PJL) *i.e.*, mPEG-*b*-PJL derived from renewable jasmine lactone monomer with free allyl groups on the backbone. Thereafter, we introduced hydroxyl, amine and carboxyl groups *via* UV mediated thiol–ene click chemistry.^30,31^ Due to the presence of free functional groups, we believe that this novel polymer could be a promising alternative to improve the drug loading content within PMs.

Thus, herein we modulated the polymer backbone to include a free functional group and investigated its effect of on drug loading capacity of the resulting micelles. Two copolymers, *i.e.*, unfunctionalized mPEG-*b*-PJL and functionalized mPEG-*b*-PJL-COOH were utilised to study the effect of ionic interactions on the encapsulation efficiency. Through utilising mPEG-*b*-PJL as a reference polymer, we investigated if the changes that occur on the drug–polymer formulation behaviour are based solely on the interaction of the active agent with the COOH pendant groups added onto the polymer backbone. We hypothesised that the electrostatic interactions between the COOH functionality on mPEG-*b*-PJL-COOH and the basic drugs increase the efficiency of entrapment compared to the respective efficiency in case of solely hydrophobic drug–polymer interactions.

Therefore, in this study, we employed various drugs with different p*K*_a_ values to prepare drug-loaded PMs. Moreover, we carried out MD simulations to gain understanding on the molecular–level interactions of the drugs with the polymers and how variations in polymer functional groups influence these interactions. The release kinetics of the anti-cancer drug sunitinib from mPEG-*b*-PJL and mPEG-*b*-PJL-COOH micelles were investigated, and the cytotoxicity of the sunitinib-loaded micelles on cancer cells was tested. Additionally, the CMC and the haemolytic capability of mPEG-*b*-PJL and mPEG-*b*-PJL-COOH were examined. Moreover, to compare the drug loading efficiency with a commercially available polymer, polyvinyl caprolactam–polyvinyl acetate–polyethylene glycol graft copolymer (Soluplus®) was chosen.^32^ Soluplus® has been developed with the objective of increasing the aqueous solubility of drugs. Soluplus® was reported to increase the aqueous solubility of various hydrophobic drugs such as estradiol, carbamazepine and griseofulvin.^31^

## Results and discussion

### Preparation and characterization of micelles

**Scheme 1:**
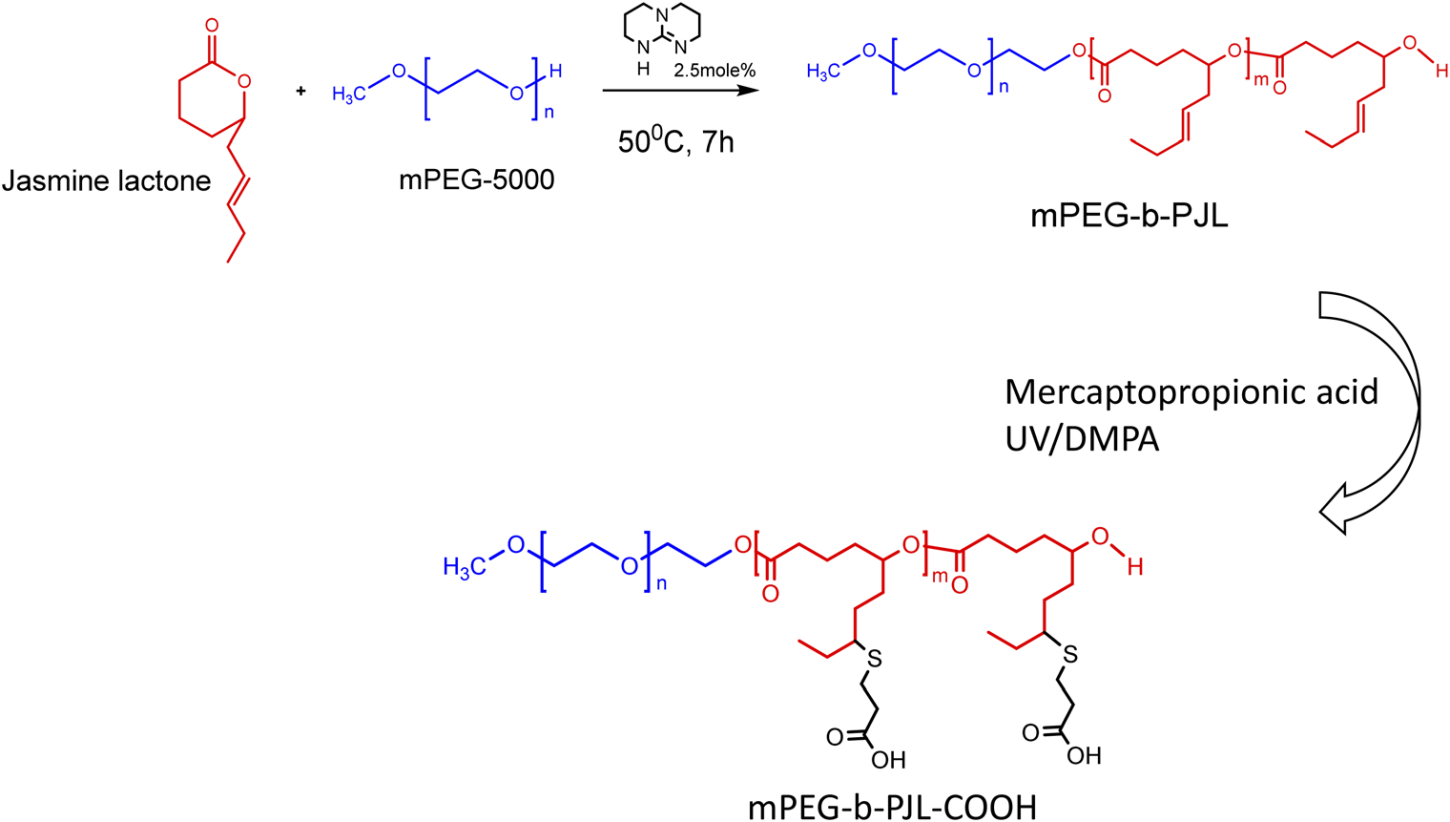
Synthesis scheme of poly(jasmine) lactone block copolymers.^39^

**Fig. 1:**
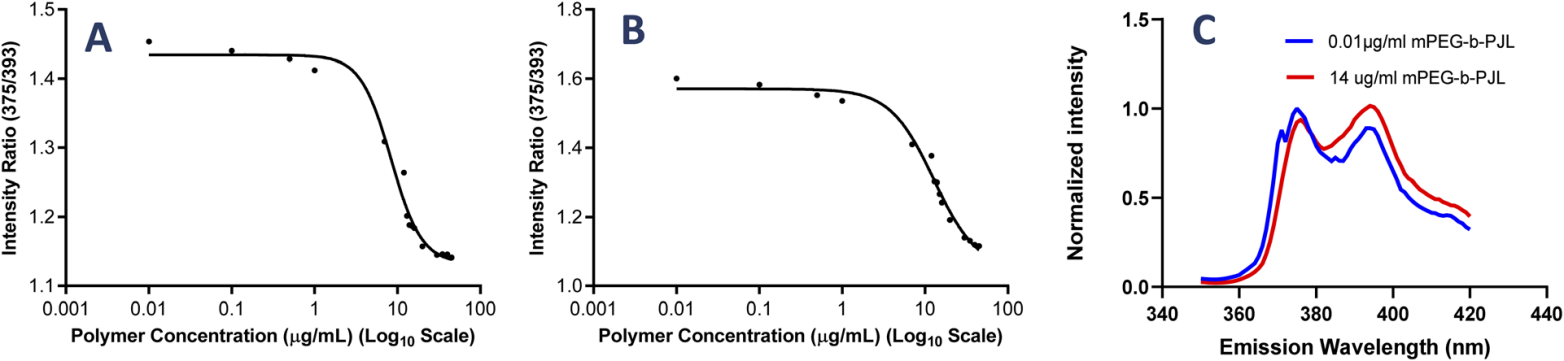
The plot between intensity ratio and polymer concentration to determine the CMC of (A) mPEG-*b*-PJL and (B) mPEG-*b*-PJL-COOH polymer. (C) Normalized fluorescence emission spectra of pyrene at different concentration of mPEG-*b*-PJL to demonstrate the shift in peak intensity at 375 and 393 nm.

The poly(jasmine lactone) polymers synthesis and characterization data are already reported in our previous publication (Scheme 1).^30^ The polymer mPEG-*b*-PJL (*M*_n_ – 8.8 kDa, dispersity – 1.4) and mPEG-*b*-PJL-COOH (*M*_n_ – 9.8 kDa, dispersity – 1.4) were used in this study to prepare micelles. The CMCs of polymers were determined through the analysis of the fluorescence spectra of the solutions containing pyrene and then calculating the pyrene 1 : 5 ratio through measuring the intensities of emitted light at 375 nm (I1) and 393 nm (I5) and plotting them against the concentration of polymer. The CMC of mPEG-*b*-PJL and mPEG-*b*-PJL-COOH was determined to be 8.4 ± 1.4 and 10.2 ± 3.9 μg mL^−1^ (Fig. 1), respectively.

The nanoprecipitation method was used to prepare empty and drug-loaded micelles for all polymers.^33^ Hydrophobic drugs used in the studies were furosemide (p*K*_a_ 3.8, a weak acid), celecoxib (p*K*_a_ 11.1, a very weak acid, practically neutral at physiological pH), carvedilol (p*K*_a_ 7.8, a weak base), sunitinib (p*K*_a_ 9.3, a weak base) and allopurinol (p*K*_a_ 10.2 a weak base); the p*K*_a_ values define the strength of their acidity or basicity.^34–38^

**Fig. 2:**
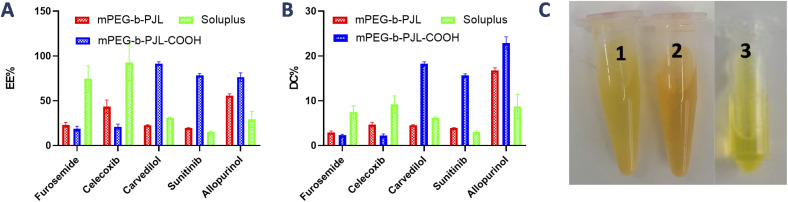
(A) Encapsulation efficiency (EE%) and (B) drug content (DC wt%) of acidic and basic drugs under study in different block copolymeric micelles. The drug in feed for the reported data in the graph is 0.5 mg mL^−1^ for furosemide and celecoxib and 1 mg mL^−1^ for carvedilol, sunitinib and 1.5 mg mL^−1^ for allopurinol. (C) Appearance of sunitinib loaded mPEG-*b*-PJL PMs (1), mPEG-*b*-PJL-COOH PMs (2) and Soluplus® PMs (3) at drug concentration of 1 mg mL^−1^.

The encapsulation efficiency and content observed with each drug in polymers are shown in Fig. 2A and B, respectively. Furosemide is a weakly acidic drug and thus negatively charged at neutral pH. Also, celecoxib is a very weakly acidic drug with the p*K*_a_ value of the sulphonamide group being near 11 and thus it is unionized at neutral pH.^40–42^ Consequently, there is no remarkable changes noticed in the drug loading efficiency based on the electrostatic factor with neither furosemide nor celecoxib. However, the entrapment efficiency of both furosemide and celecoxib is slightly better with mPEG-*b*-PJL micelles which could be due to the dominance of hydrophobic interactions (Fig. 2 and Table 1). On the other hand, due to the slightly basic character of carvedilol, sunitinib and allopurinol, electrostatic attraction between the negatively charged COOH groups of the polymer and the positively charged drugs was observed. This led to high drug loading efficiency in mPEG-*b*-PJL-COOH micelles. The remarkable entrapment efficiency (EE) mPEG-*b*-PJL-COOH copolymer at drug concentration (in feed) of 0.5 mg mL^−1^ for basic drugs (Fig. S1 and Table S1†) prompted us to increase the drug feed to 1 mg mL^−1^ which maintained superior EE% as shown in (Fig. 2 and Table 1). This experiment suggested that the drug in feed played an important role accounting for the overall drug content in a carrier.

**Table 1:** Characterization data of PMs prepared from block copolymers of mPEG-*b*-PJL, mPEG-*b*-PJL-COOH and Soluplus® (d nm^−1^ – hydrodynamic diameter in nanometers, SD – Standard deviation, PdI – polydispersity index-drug content in wt% – encapsulation efficiency% – critical micelle concentrations mPEG-*b*-PJL and mPEG-*b*-PJL-COOH)

Sample	Size by volume (peak 1) (d nm^−1^) (±SD)	*Z*-average size (d nm^−1^) (±SD)	PdI + SD	Drug content wt% ± SD	EE% ± SD
mPEG-PJL	62.26 ± 2.34	62.48 ± 1.65	0.32 ± 0.01		
mPEG-PJL-COOH	46.59 ± 1.11	59.51 ± 1.39	0.46 ± 0.03		
Soluplus®	104.17 ± 1.44	83.53 ± 0.8	0.21 ± 0.01		
Furosemide loaded mPEG-PJL (0.5 mg mL^−1^)	77.5 ± 2.46	61.48 ± 1.75	0.19 ± 0.01	2.9 ± 0.37	23 ± 2.9
Furosemide loaded mPEG-PJL-COOH (0.5 mg mL^−1^)	78.75 ± 3.02	62.29 ± 2.36	0.24 ± 0.03	2.3 ± 0.21	18.6 ± 1.69
Furosemide loaded Soluplus® (0.5 mg mL^−1^)	107.1 ± 3.8	84.93 ± 0.58	0.195 ± 0.01	7.45 ± 1.44	74.57 ± 14.44
Celecoxib loaded mPEG-PJL (0.5 mg mL^−1^)	46.57 ± 3.99	44.97 ± 3.64	0.21 ± 0.04	4.66 ± 0.52	43.4 ± 7.22
Celecoxib loaded mPEG-PJL-COOH (0.5 mg mL^−1^)	76.71 ± 0.87	54.26 ± 0.57	0.24 ± 0.003	2.24 ± 0.35	20.8 ± 3.17
Celecoxib loaded Soluplus® (0.5 mg mL^−1^)	131 ± 18.2	93.02 ± 3.04	0.216 ± 0.01	9.24 ± 1.99	92.45 ± 19.98
Carvedilol loaded mPEG-PJL (1 mg mL^−1^)	76.31 ± 1.36	62.1 ± 0.11	0.17 ± 0.01	4.5 ± 0.1	22.68 ± 0.49
Carvedilol loaded mPEG-PJL-COOH (1 mg mL^−1^)	77.61 ± 4.32	72.47 ± 0.41	0.26 ± 0.02	18.29 ± 0.42	91.49 ± 2
Carvedilol loaded Soluplus® (1 mg mL^−1^)	147.57 ± 14.16	128.93 ± 3.42	0.23 ± 0.02	6.18 ± 0.22	30.9 ± 0.73
Sunitinib loaded mPEG-PJL (1 mg mL^−1^)	95.34 ± 1.95	78.54 ± 0.78	0.2 ± 0.01	4 ± 0.09	16.67 ± 0.47
Sunitinib loaded mPEG-PJL-COOH (1 mg mL^−1^)	61.05 ± 0.39	45.78 ± 0.21	0.22 ± 0.01	15.16 ± 0.39	75.83 ± 1.97
Sunitinib loaded Soluplus® (1 mg mL^−1^)	156.7 ± 6	129.34 ± 4.02	0.31 ± 0.002	2.96 ± 0.3	14.82 ± 1.5
Sunitinib loaded P123 (1 mg mL^−1^)				2.31 ± 0.05	11.54 ± 0.25
Allopurinol loaded mPEG-PJL (1.5 mg mL^−1^)	50.36 ± 2.51	43.74 ± 1	0.19 ± 0.01	16.75 ± 0.6	55.84 ± 1.99
Allopurinol loaded mPEG-PJL-COOH (1.5 mg mL^−1^)	79.72 ± 3.69	63.33 ± 0.67	0.23 ± 0.003	22.89 ± 1.4	76.31 ± 4.82
Allopurinol loaded Soluplus® (1.5 mg mL^−1^)	155 ± 7.12	122.53 ± 2.15	0.23 ± 0.01	8.72 ± 2.72	29,08 ± 9.07

As expected, sunitinib loaded mPEG-*b*-PJL-COOH micelles at drug concentration 1 mg mL^−1^, showed outstanding improvement in drug content (DC)% and EE% compared to sunitinib-loaded mPEG-*b*-PJL micelles. The difference in loading was evident with sunitinib due to its colour. The visual inspection of samples clearly demonstrates higher drug loading with mPEG-*b*-PJL-COOH with a remarkably darkened orange colour solution compared to light yellow with mPEG-*b*-PJL (Fig. 2C). For drug-loaded Soluplus® micelles at 1 mg mL^−1^ drug concentration of the basic drugs under study, drug content and encapsulation efficiency were notably lower than those in mPEG-*b*-PJL-COOH and slightly lower than those in mPEG-*b*-PJL. The results clearly demonstrate that free functional groups on polymers can interact with oppositely charged drugs and consequently increase the drug loading efficiency. Indeed, the increased negative charge in mPEG-*b*-PJL-COOH compared to mPEG-*b*-PJL was clearly observed in surface zeta potential; mPEG-*b*-PJL and mPEG-*b*-PJL-COOH zeta potentials were −3.63 ± 1.41 and −16.6 ± 1.9 mV, respectively, at neutral pH. The zeta potential of sunitinib loaded micelles was also observed to be slightly negative where mPEG-*b*-PJL and mPEG-*b*-PJL-COOH zeta potentials were −11.0 ± 1.69 and −15.6 ± 2.0 mV, respectively (Fig. S2†). The utilized polymers were able to enhance the aqueous solubility of the hydrophobic drugs with different degrees. Whereas, mPEG-*b*-PJL enhanced the aqueous solubility for furosemide, celecoxib, carvedilol, sunitinib and allopurinol by 6, 43.4, 384, 16.6 and 6 times, respectively. While mPEG-*b*-PJL-COOH enhanced the aqueous solubility of the same drugs by 5, 20.8, 1554, 75.8 and 8 times, respectively. Finally, with Soluplus®, the aqueous solubility increased by 20.7, 92.46, 525.5, 14.8 and 3 times, respectively.^43–47^ The increment in aqueous solubility of the basic drugs loaded into mPEG-*b*-PJL-COOH PMs was found to be higher compared to other two polymers. This result supports the previous work, which suggest that not only the length of the hydrophobic chain greatly influence the solubilization capacity of PMs but the type of functionality present also does.^48^ However, good performance of Soluplus® was observed in the case of furosemide and celecoxib. To understand the reason behind this, we performed MD simulation studies to reveal the dominant drug–polymer interaction (see Computational studies).

Next, we performed hydrodynamic size analysis to establish that these micelles are of nanometer size range. The *Z*-average, PdI and the hydrodynamic size analysis of the volume distribution curve of mPEG-*b*-PJL, mPEG-*b*-PJL-COOH and Soluplus® loaded and unloaded micelles were determined by DLS. DLS suggested that the majority of block copolymer micelles of mPEG-*b*-PJL and mPEG-*b*-PJL-COOH and their loaded micelles lie in the range of 45–100 nm while Soluplus® micelles lie in the range of 100–160 nm along with some larger aggregates representing the second peak of the DLS graph (Table 1) (Fig. S3†). mPEG-*b*-PJL and mPEG-*b*-PJL-COOH blank micelles show noticeable second peak that almost disappears after drug loading. This phenomenon could be a result of higher levels of aggregates in blank micelles that weakens after drug encapsulation due to favourable interactions (hydrophobic and/or electrostatic) between the drug and the polymer.^49^ Moreover, there is a slight surge in size noticed in most drug-loaded mPEG-*b*-PJL and mPEG-*b*-PJL-COOH micelles after drug loading. The size distribution of Soluplus® blank micelles is similar to previously reported results with acyclovir and rifampicin. Besides, the slight increase in the size of the drug loaded Soluplus® micelles compared to its blank micelles was also noticed in these previous studies.^50,51^ The increase in size with drug encapsulation could be related with the dynamic nature of the PMs since the encapsulation of the hydrophobic drug within the core of the micelles may modify the aggregation behaviour and may result in larger micellar sizes.^52^

### Computational studies

The physico-chemical properties of a polymer have a huge impact on the nature of non-bonding interactions with various drug molecules. Polymer molecules having favourable interactions with the small drug molecule tend to increase the overall solubility of that drug by avoiding drug–drug interactions and replacing them with polymer–drug interactions. The strong polymer–drug interactions counterbalance the drug–drug interactions, restricting the drug molecule from forming a crystalline form, thus increasing the stability to the formulation. Hence, predicting polymer–drug interactions is of great importance for envisaging the right polymer–drug combination to achieve a high solubility as well as stability.

The non-bonding interactions of polymers with small molecules may include hydrogen bonding, van der Waals (vdW) and coulombic interactions. The objective of molecular dynamics (MD) simulations in this work is to understand the dominant forces responsible for high drug loading. Moreover, an optimized MD simulation protocol could save time in future studies involving PJL-based copolymer micelles aiming for solubility enhancement.

#### Hydrogen bonding

The presence of intermolecular hydrogen bonding promotes the miscibility of polymer-based drug formulations.^53,54^ Hydrogen bond formation involves a transfer of a charge from a proton acceptor to a proton donor and a change in local environment, which is often influenced by the acidity and basicity of the donor and the acceptor atoms, the rigidity of the polymer and stereochemical factors.^55^ Though the strength of a hydrogen bond is weaker than that of a covalent bond, it is much stronger than vdW interactions. The typical strength of a hydrogen bond varies over a range of 5–35 kcal mol^−1^ and is also influenced by the bond angle.^56^

**Fig. 3:**
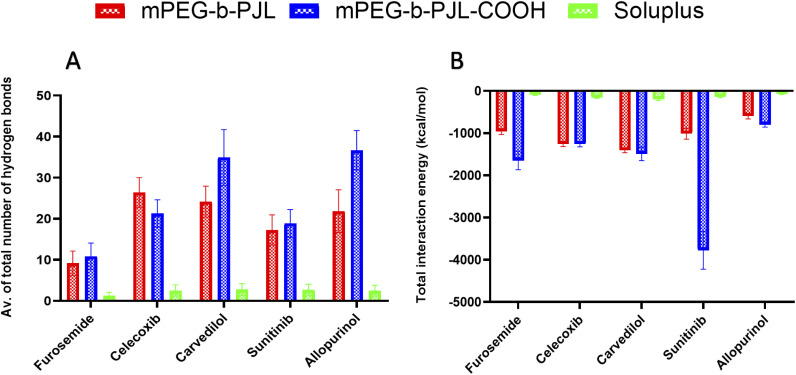
(A) Average number of hydrogen bonds formed between drug molecules and the mPEG-*b*-PJL, mPEG-*b*-PJL-COOH or Soluplus® polymers during a 500 ns-long molecular dynamics (MD) simulation. The values are averaged over the entire simulation trajectory. (B) The total interaction energy calculated as the sum of electrostatic and van der Waals interactions between the different polymers and drug molecules during a 500 ns-long MD simulation. The values are averaged over the entire simulation trajectory.

We simulated and registered the hydrogen bond interactions taking place between mPEG-*b*-PJL, mPEG-*b*-PJL-COOH or Soluplus® and a selected set of drug molecules. Fig. 3A shows the average number of drug–polymer hydrogen bond interactions. During the MD simulation with mPEG-*b*-PJL, the greatest number of hydrogen bonds were observed for celecoxib, allopurinol, and carvedilol. The sunitinib molecules showed an intermediate number of hydrogen bonds. Among all, furosemide showed the least hydrogen bonds with the polymer. Similarly, in mPEG-*b*-PJL-COOH simulation, allopurinol and carvedilol showed the significantly highest number of hydrogen bonds, followed by sunitinib and celecoxib. Comparatively, a lesser hydrogen bond network formation was observed for furosemide. When these interactions were compared with Soluplus®, carvedilol, sunitinib, celecoxib and allopurinol showed a good hydrogen bond network while furosemide showed poor interactions.

### van der Waals (vdW) and coulombic interactions

Apart from the number of hydrogen bond interactions, we calculated the vdW and coulombic interactions taking place between the polymers and the drug molecules. The total interaction energy in kcal mol^−1^ was then calculated as a sum of both vdW and coulombic interactions as shown in Fig. 3B. The more negative values mean more favourable interactions and the more positive ones *vice versa*.

In case of mPEG-*b*-PJL, the interactions were stronger for carvedilol and celecoxib, followed by sunitinib and furosemide. Weak interactions were seen for allopurinol. In mPEG-*b*-PJL-COOH polymer, the presence of the acidic functional group showed much more stronger interactions with equally basic moieties on sunitinib, resulting in a more favourable total interaction energy as compared to the other drug molecules. Furosemide, carvedilol and celecoxib also showed significantly strong total interaction energies, while for allopurinol the total interaction energy was the least favourable of all.

For Soluplus® the total interaction energy was in the following order from more favourable to least favourable interactions starting with carvedilol, celecoxib, sunitinib, furosemide and allopurinol at the end.

Overall, the MD simulation results for mPEG-*b*-PJL suggest abundant hydrogen bonding as well as strong vdW and coulombic interactions for both celecoxib and carvedilol. Though allopurinol also shows a comparative number of hydrogen bonds it had a less favourable interaction energy than these two drug molecules. In case of mPEG-*b*-PJL-COOH polymer based on both total interaction energy and hydrogen bonding results, both carvedilol and sunitinib showed the most favourable interactions followed by allopurinol and celecoxib, while furosemide showed the weakest interactions. On other hand, the drug interactions with Soluplus® were observed to follow the order: carvedilol > celecoxib > sunitinib > allopurinol > furosemide.

When both mPEG-*b*-PJL and mPEG-*b*-PJL-COOH are compared with respect to the total number of hydrogen bonds and total interaction energy with these five drug molecules, except the number of hydrogen bonds formed between celecoxib and mPEG-*b*-PJL, mPEG-*b*-PJL-COOH polymer was able to form more or stronger interactions with all other drug molecules.

### *In vitro* release behaviour and cytotoxicity of sunitinib from block copolymer micelles

Sunitinib is soluble in acidic aqueous solutions (25 mg mL^−1^ at pH 1.2–6.8). Regardless, the solubility of the drug rapidly decreases at pH values greater than 6.8 and it is very poorly soluble in water and thus, sunitinib is classified as a low-solubility compound according to the biopharmaceutics classification system (BCS).^57,58^ Additionally, the poor dissolution rate of the drug results in poor *in vivo* bioavailability after oral administration with maximum reported plasma concentration of 20–30 ng mL^−1^ reached in 5–7 h.^46^ Therefore, utilising the nanotherapeutic strategy to deliver hydrophobic chemotherapeutic agents is a powerful approach to overcome current drug delivery challenges. PMs solubilize the drugs and enhance their stability, which allows for longer circulation times to be reached.^59^ Therefore, in order to assess the potential of the new polymer in drug delivery, we firstly studied the release patterns of sunitinib from mPEG-*b*-PJL and mPEG-*b*-PJL-COOH micelles followed by cytotoxicity of sunitinib-loaded micelles on cancer cells.

**Fig. 4:**
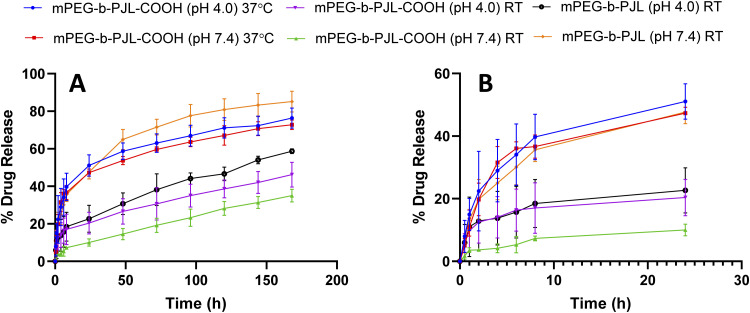
(A) *In vitro* release pattern of sunitinib from mPEG-PJL and mPEG-PJL-COOH block copolymer micelles at two different pHs using PBS pH 7.4 and acetate buffer pH 4 as release medias at 25 °C and 37 °C (B) zoom in to the first 24 h release pattern.

The release pattern of sunitinib was examined at different pH and temperatures for 7 days and the results are presented in Fig. 4. Initial burst release of sunitinib was noticed from mPEG-*b*-PJL micelles with 35.6% and 18% release in the first 8 h at pH 7.4 and pH 4, respectively. After 7 days, 85.16% and 58.74% release of sunitinib was observed at pH 7.4 and pH 4, respectively at room temperature (RT). The more extended-release pattern in the acetate buffer (pH 4) could be prompted by the acid catalysed hydration of mPEG-*b*-PJL unsaturated bond.^60^ This could lead to alteration of the physico-chemical properties of the polymer. On the other hand, the release of sunitinib from mPEG-*b*-PJL-COOH at pH 7.4 exhibited a slower and more extended release with only 7.3% at 8 h and only 35.05% release after 7 days. This slow release could be attributed to the strong ionic interaction between the acidic polymer and the basic drug, thus hindering the release from the micellar core. In contrast to the release profile observed from the mPEG-*b*-PJL micelles, the initial release rate of sunitinib from mPEG-*b*-PJL-COOH was faster in acetate buffer compared to its initial release rate in PBS reaching an initial release of 17.04% after 8 h and then achieving a more sustained rate up to 46.24% at 7 days. The acidic pH promotes the protonation of the acidic carboxylic acid, which may aid in overcoming the strong ionic interactions between the drug and the polymer, thus enhancing the drug release compared to the neutral pH. Earlier, Ying-Hsia Shih *et al.* demonstrated sunitinib release from mPEG-PCL micelles where 85% of the sunitinib had been released within 72 h in PBS at 37 °C.^61^ We here successfully demonstrate a more sustainable drug release pattern of sunitinib from PJL-based micelles.

Kost *et al.* also reported similar results where polymer with free –COOH groups demonstrated 25% drug release after 1 h in physiological conditions (pH = 7.4, 37 °C). In contrast, non-functionalized polymer released 75% of loaded drug after 1 h in similar conditions.^62^ The authors attributed the slower release with functionalized polymers to the electrostatic interaction between the negatively charged carboxyl moieties of NPs with the positively charged amino group of the drug (doxorubicin). Rezaian *et al.* made a similar observation with DOX and carboxyl functionalized fullerenes. Their MD simulation study showed that the total interaction energies are significantly more favourable at neutral pH than at acidic pH. Furthermore, the main interaction between DOX and carboxyl functionalized fullerene was shown to be electrostatic while van der Waals interactions had almost no role.^63^

The release experiment was repeated for sunitinib loaded mPEG-*b*-PJL-COOH micelles at 37 °C ± 0.5 to understand the effect of temperature. The release rate of sunitinib was enhanced and no significant difference was observed in the release rate based on the pH at 37 °C. These results suggested that temperature has a strong influence on release compared to pH. PJL is found to be an amorphous polymer as the DSC analysis of mPEG-*b*-PJL and mPEG-*b*-PJL-COOH showed a *T*_g_ of −56.06 °C and −49.58 °C, respectively. However, due to the presence of PEG in the structure, melting point were also observed for the polymer’s mPEG-*b*-PJL and mPEG-*b*-PJL-COOH, at 55.20 °C and 44.50 °C (Fig. S4†). Since the drug release mechanism here is diffusion based and thus, due to the low melting point and amorphous nature of one block, an accelerated drug release at 37 °C was observed. This could be attributed to the increase in chain flexibility with increase in the temperature, which results in faster diffusion/sneaking of drug independent to pH. In previous studies, micelles based on amorphous polymers also demonstrate faster release compared to semicrystalline polymers.^9,17^

*In vitro* cytotoxicity assessment on cancer cells is a crucial experiment to evaluate the potential activity and toxicity of novel drug carriers. In this work, the blank and sunitinib loaded mPEG-*b*-PJL and mPEG-*b*-PJL-COOH micelles were evaluated for their cytotoxicity on HeLa cells. Pluronic P123 (P123) micelles were also prepared and used as reference in the cell studies. The P123 blank and sunitinib loaded micelles were prepared in a similar fashion and the drug content found in these micelles were 2.31 ± 0.05, w/w%. P123 (EO_20_-PO_68_-EO_20_) is a common polymer that is extensively investigated as a carrier in drug delivery.^64^ Similar to PJL based micelles, P123 also contains polyethylene oxide (PEO) with a total molecular weight of 5750 g mol^−1^ containing 30% of PEO in P123 copolymer.^65^ Many of the Pluronic block copolymers are listed in U.S. Pharmacopoeia and are approved for various medical applications as formulation excipients.^66^ Due to the extensive difference in molecular weight of Soluplus® and PJL based copolymer, we decided to exclude them from cell studies but instead used P123 as a more relevant reference polymer.

**Fig. 5:**
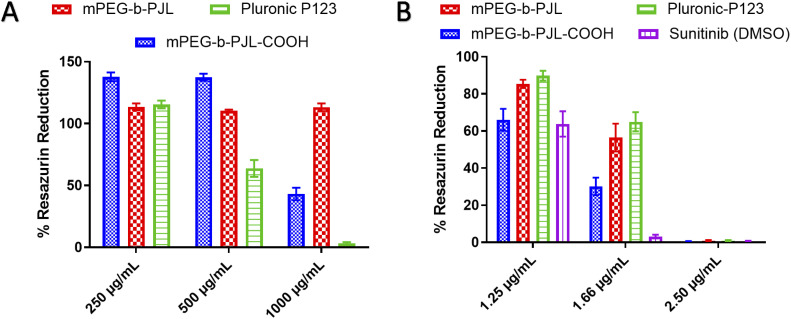
Effect of micelle formulations (A) blank micelles and (B) Sunitinib loaded micelles, on HeLa cells proliferation when incubated for 72 h with different concentrations of formulation (*n* = 3).

The toxicity profiles of the utilised polymers mPEG-*b*-PJL, mPEG-*b*-PJL-COOH and P123 were evaluated using Alamar Blue cell viability assay. Blank mPEG-*b*-PJL micelles were found to be non-toxic up to tested 1 mg mL^−1^ of polymer concentration (Fig. 5A) which suggests good tolerability of the polymer. On the other hand, mPEG-*b*-PJL-COOH at lower concentrations up to 0.5 mg mL^−1^ demonstrate a noticeable increase in cell viability compared to control groups. However, a sharp decline in cell viability was observed where only ∼43% viable cells were present at concentration of 1 mg mL^−1^. These results revealed that free functional groups (here COOH) have a direct impact on cell viability. The COOH terminated polymer is found to be more toxic compared to non-functionalized polymer most likely due to the ionic interaction between carboxylic groups and cells.^67,68^ P123 showed no clear toxicity at concentration of 0.25 mg mL^−1^, however, cell viability declined as P123 increased with almost no viable cell at concentration 1 mg mL^−1^ suggesting that P123 is more toxic than PJL based micelles at higher concentration. The toxicity of P123 could be related to its inherent cytotoxicity and its ability to inhibit P-glycoprotein (P-gp) drug efflux transport system. As a result, the inhibition of the P-gp drug efflux transport system that is overexpressed in cancer cell lines leads to improved cytotoxicity. Moreover, Pluronic copolymers exhibited better antimetastatic effects.^69^

In terms of sunitinib loaded micelles, we used three concentration of drug and based on the loading, even the higher drug concentration sample contains polymer less than 250 μg mL^−1^. As presented in Fig. 5B, Sunitinib loaded in mPEG-*b*-PJL and P123 micelles were found to be less toxic compared to free drug and mPEG-*b*-PJL-COOH micelles at lower concentration. However, all formulations are equally toxic at 2.5 μg mL^−1^ concentration suggesting dose dependent toxicity. Interestingly, sunitinib loaded in mPEG-*b*-PJL-COOH were found to be more toxic to the cells compared to its counterparts, which might be due to the higher drug content leading to the faster release of drug inside the cells. In contrary, the sustained release of the drug from the micelles can be attributed to the lower toxicity exerted by micelles formulations compared to free drug.

### *Ex vivo* haemolytic study

The sunitinib-loaded mPEG-*b*-PJL and mPEG-*b*-PJL-COOH micelles showed promising results *in vitro* for their further development in drug delivery; nevertheless, the toxicity of nanoparticles can sometimes pose a challenge to their application *in vivo*. Therefore, haemolysis study was performed for blank mPEG-*b*-PJL and mPEG-*b*-PJL-COOH micelles to assess their blood compatibility upon injection. The biocompatibility of pharmaceutical excipients is an important consideration during formulation development in which haemolysis rate for the drug carrier below 10% is considered non-haemolytic, while values higher than 25% holds risk for haemolysis.^70^

**Fig. 6:**
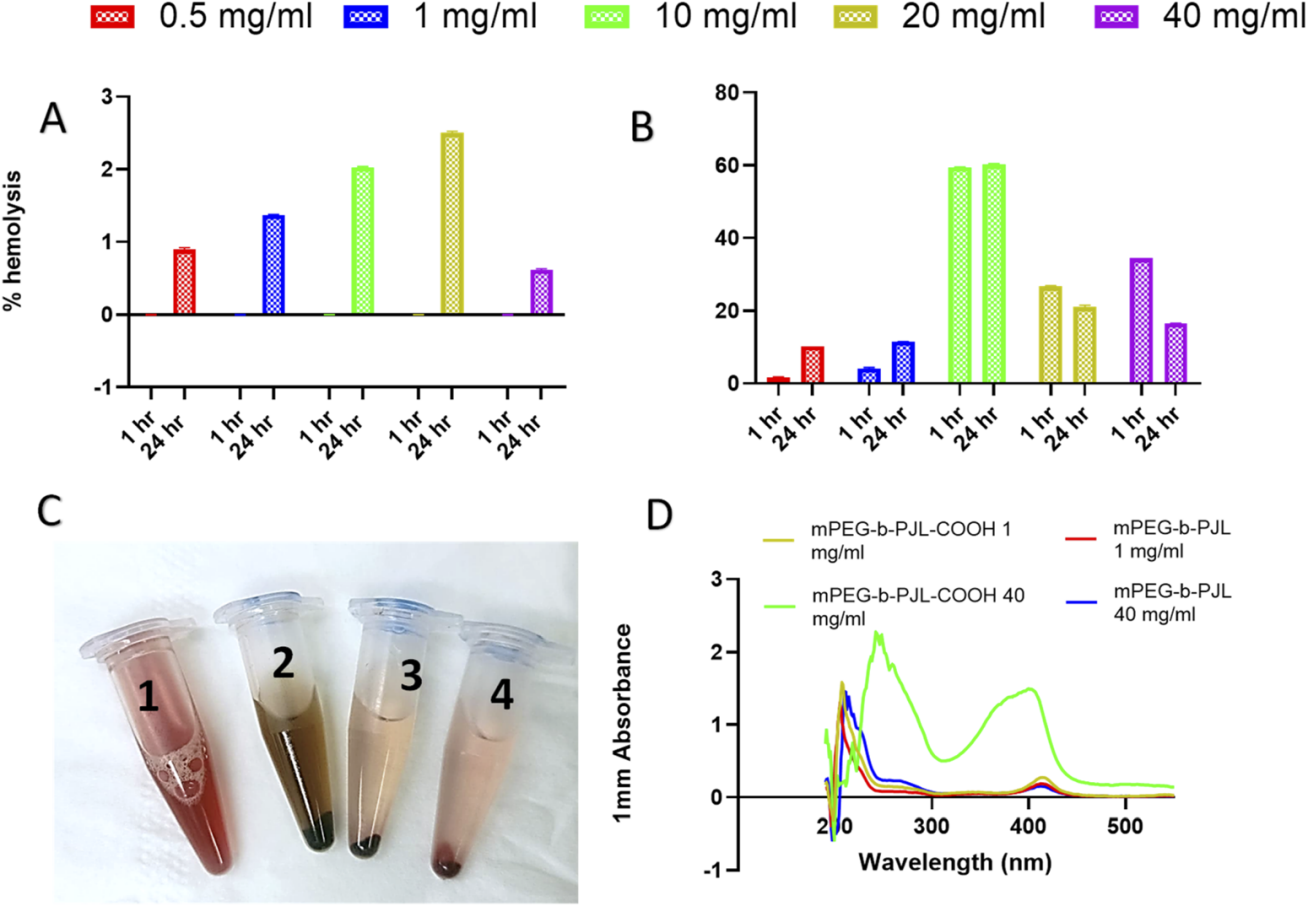
(A) The haemolysis percentage observed with different concentration of mPEG-PJL micelles incubated for 1 h and 24 h at 37 °C. (B) The haemolysis percentage observed with different concentration of mPEG-PJL-COOH micelles incubated for 1 h and 24 h at 37 °C (C) Appearance of tubes incubated for 24 h after centrifugation number 1, 2, 3 and 4 represents positive control sample, sample treated with 40 mg mL^−1^ mPEG-PJL-COOH micelles, sample treated with 40 mg mL^−1^ mPEG-PJL micelles and negative control sample respectively. (D) UV-spectra of haemolysis rate samples of different polymer concentrations.

The mPEG-*b*-PJL and the mPEG-*b*-PJL-COOH micelles were tested for their haemolytic capacity up to the concentration of 40 mg mL^−1^ in static conditions. As shown in Fig. 6C, visual inspection of the incubated tubes after centrifugation provided a preliminary idea of RBCs lysis. The absence of red colour observed in supernatant in mPEG-*b*-PJL micelles samples even at 40 mg mL^−1^ concentration suggested negligible toxicity of this copolymer. No haemolysis was observed at 1 h incubation while the maximum haemolysis noted for mPEG-*b*-PJL micelles after 24 h incubation is not exceeding 3% (Fig. 6A). These results promote mPEG-*b*-PJL as a safe polymer for intravenous injection. In contrast to the general phenomena, the haemolysis rate was dropped at 40 mg mL^−1^ concentration compared to 20 mg mL^−1^.

In case of mPEG-*b*-PJL-COOH micelles, a significant amount of lysis was observed at 10 mg mL^−1^ concentration after 1 h incubation but was below 10% at 1 mg mL^−1^ concentration.

A noticeable increase in haemolysis was also observed after 24 h incubation where even lower concentrations (0.5 mg mL^−1^ and 1 mg mL^−1^) slightly exceeded the non-haemolytic cut-off percentage of 10%. The results suggested that presence of free anionic groups (–COOH) on polymers has the potential to increase the haemolysis rate to several folds. This toxicity could be explained by the repellent forces between carboxylic acid groups which leads to less tendency to self-assembly.^71^ Also, in one study carried out by Muranaka *et al.* it was confirmed that COOH groups are responsible for haemolysis activity and compounds without COOH functional groups showed no haemolysis activity. In other words, out of eight compounds, seven sapogenins were haemolytic owing to possessing COOH groups in their structures.^72^ In an another study conducted by Lavasanifar *et al.*, block copolymers with end and pendant carboxyl groups were analysed. mPEO-*b*-PCCL showed haemolysis activity due to pendant COOH groups while mPEO-*b*-PBCL with end COOH groups was not toxic.^73^ Nevertheless, inside the human body, the concentration of micelles would be diluted with approximately 5 L of blood and thus the amount of micelles interacting with RBCs would be greatly diluted which will most probably increase the non-toxic window of the polymer.^9^

Additionally, there was a sharp decline in haemolysis rate in concentrations higher than 10 mg mL^−1^ of mPEG-*b*-PJL-COOH micelles and the decline became more prominent after 24 h incubation (Fig. 6B). It might be accompanied with a slight shift in *λ*_max_ of haemoglobin (Fig. 6D). The phenomenon is probably related to the colour change previously noticed through visual inspection with 20 and 40 mg mL^−1^ which indicates a stronger interaction between the mPEG-*b*-PJL-COOH and the RBCs at higher concentrations (Fig. 6). This observation could be explained through the work conducted by Hasan *et al.* under stress conditions, haemoglobin (Hb) forms aggregates which leads to loss of heme and thus they utilised varying acid concentrations in the range of 0–60 mM to study conformational transition in Hb, structural loss and finally aggregation of protein. The secondary structure of the Hb molecule consists mainly of α-helix and the presence of acid leads to the transformation of the native helical structure to a non-native β-sheet structure *via* intermediate formation causing aggregation of Hb. Gradual loss of alpha content occurred as the acid concentration increased because of the α-helical structure perturbation and the tendency to shift towards β-sheet. At 50 mM of both acids, comprehensive loss of all helical structure and altering to majorly β-sheets was observed which indicated intermolecular β-sheets formation because of aggregates formed.^74^ Thus, the decreased absorbance accompanied by a shift in *λ*_max_ observed as the concentration of mPEG-*b*-PJL-COOH increased may not indicate an actual decrease in haemolysis rate. However, the structural disruption of the haemoglobin released from lysed RBCs induced by the COOH-pendant renders the UV absorption spectrum of haemoglobin an unreliable methodology for measuring its concentration.

## Conclusions

In this study, we explored the effect of modulation of the polymer backbone to include a free functional group on the drug loading capacity of micelles through utilizing mPEG-*b*-PJL and its carboxyl post-functionalized counterpart mPEG-*b*-PJL-COOH. We demonstrated that the electrostatic interactions between the COOH pendant on mPEG-*b*-PJL-COOH and a range of basic drug molecules enhances efficiency of entrapment in a fashion that may surpass that of the hydrophobic interactions. MD simulations were carried out to understand the molecular interactions that are responsible for the increased or decreased overall drug loading. We observed that both hydrogen bonding interactions along with van der Waals and electrotactic interactions are more favourable between the basic drugs and mPEG-*b*-PJL-COOH than mPEG-*b*-PJL. The release of sunitinib from micelles was found to be pH and temperature dependent. Sunitinib-loaded micelles exhibited a dose-dependent toxicity on cancer cells. *Ex vivo* haemolytic study showed mPEG-*b*-PJL micelles to be non-haemolytic up to a concentration of 40 mg mL^−1^; however, mPEG-*b*-PJL-COOH micelles at concentrations higher that 1 mg mL^−1^ were shown to cause haemolysis that was derived by anionic COOH pendant groups. Also, a noticeable decrease in absorbance was observed at higher polymer concentrations. To better understand this phenomenon, our future studies are aimed at performing elaborate haemolysis studies on these polymers. Thus, the results of this study suggest that functionalization of a polymer to introduce certain molecular forces that influence drug–polymer interaction is an effective approach to improve drug loading capacity and tune the release rate from PMs.

## Author contributions

Conceptualization, K.K.B; methodology, K.K.B., R.B.; investigation, K.K.B., Aliaa.A., Afshin.A.A., and R.B.; data curation, K.K.B., R.B.; writing – original draft preparation, Aliaa.A., Afshin.A.A, R.B., and K.K.B; writing – review and editing, O.S.A., J.M.R; resources and supervision, O.S.A., C.E.W., and J.M.R. All authors have read and agreed to the published version of the manuscript.

## Conflicts of interest

The authors declare no conflict of interest.

## Supplementary Material

RA-012-D2RA03962A-s001
