# Supplementary material for: Functional block copolymer micelles based on poly (jasmine lactone) for improving the loading efficiency of weakly basic drugs
Source: RSC Adv. 2022 Sep 21;12(41):26763–75. doi: 10.1039/d2ra03962a (PMC9490767; doi:10.1039/d2ra03962a)
Supplement: RA-012-D2RA03962A-s001 [file RA-012-D2RA03962A-s001.pdf]

Electronic Supplementary Material (ESI) for RSC Advances.
This journal is © The Royal Society of Chemistry 2022

Supporting Information

Functional block copolymer micelles based on poly (jasmine lactone) for improving the loading efficiency of weakly basic drugs

Aliaa A. Ali[1], Rajendra Bhadane[1,2], Afshin Ansari Asl[1,3], Carl-Eric Wilén[3], Outi Salo-Ahen[1,2], Jessica M. Rosenholm*[1], and Kuldeep Kumar Bansal *[1,3]

[1]Pharmaceutical Sciences Laboratory, Faculty of Science and Engineering, Åbo Akademi University, BioCity (3rd floor), Tykistökatu 6A, 20520 Turku, Finland
[2] Structural Bioinformatics Laboratory, Faculty of Science and Engineering, Biochemistry, Åbo Akademi University, 20520 Turku, Finland
[3]Laboratory of Molecular Science and Engineering, Åbo Akademi University, Aurum, Henrikinkatu 2, 20500 Turku, Finland
*Correspondence: Jessica.rosenholm@abo.fi, kuldeep.bansal@abo.fi

Experimental

Materials

Furosemide purchased from Fargon, Allopurinol (>98%), Celecoxib (>99%) and Sunitinib, Free base (>99%) purchased from LC laboratories. Carvedilol (Pharmaceutical Secondary Standard; Certified Reference Material), and solvents, methanol for HPLC (≥99.9%), acetone for HPLC (≥99.8%) have been purchased from Sigma-Aldrich and used as received. Soluplus® was purchased from BASF, Germany. MilliQ® water was used throughout the study.

Micelles preparation and characterization

Polymer synthesis

mPEG-b-PJL polymer have been synthesized according to method in[1] via ring opening polymerization where the amphiphilic block copolymer of PJL was prepared at 50 °C using methoxy(polyethylene glycol) (mPEG) as the initiator and 1,5,7-triazabicyclo[4.4.0]dec-5-ene (TBD) as catalyst in the absence of solvents. mPEG-PJL-COOH was synthesized through post-functionalization of mPEG-b-PJL via UV light induced thiol-ene click reaction[1](Scheme 1).

Determination of critical micelle concentration (CMC) of mPEG-b-PJL and mPEG-b-PJL-COOH

The CMC of mPEG-b-PJL and mPEG-b-PJL-COOH was determined via the method reported in[1]. In brief, pyrene stock solution of 6×10^{-7} M in acetone was prepared and a pre-calculated quantity of pyrene was transferred into vials and then the acetone was left to evaporate at room temperature in dark. A range of concentrations (from 0.001 to 50 µg/mL) of polymer solution in water were then added to each vial and left overnight in the dark under agitation to equilibrate. The fluorescence spectra of the solutions were analysed in the range of 350 to 450 nm at an excitation wavelength of 335 nm on PC1 photon counting spectrofluorometer. The pyrene 1:5 ratio was calculated by measuring the intensities of emitted light at 375 nm (I1) and 393 nm (I5) and then plotted against the concentration of polymer used (log scale). The resulting curve was fitted using nonlinear regression (sigmoidal, 4PL, X axis log scale) using GraphPad Prism (version 8.4.2) software to calculate the CMC value in which the inflection point of the sigmoidal curve was considered as the CMC value of the polymer.

Micelles preparation

mPEG-b-PJL, mPEG-b-PJL-COOH and Soluplus® drug-loaded PMs were prepared simultaneously for each drug under the same conditions using a single-step nano-precipitation method with minor modifications[2]. Briefly, Furosemide (0.5 mg) was dissolved along with the polymer (5mg) in acetone

(0.5 mL) and added drop wise into Milli-Q water (1 mL) under stirring (1000 rpm). The solution was then stirred overnight at room temperature and left (open vial) to ensure the complete removal of organic solvent. Celecoxib (0.5mg), Carvedilol (0.5mg), Carvedilol (1mg) and Sunitinib (1mg) micelles were synthesized using same procedures. Empty micelles were prepared using same procedure without drug. Allopurinol (1.5 mg) was dissolved along with 5(mg) of polymer in 2 ml of acetone: methanol mixture (1:4), sonicated, before adding in water due to its poor solubility in acetone.
Next, the micelles were transferred to an eppendorf tube and centrifuged at 13500 rpm for 15 mins and then the supernatant was collected for further characterization.

Drug content

Suitable amount of drug-loaded micelles was withdrawn and diluted with methanol in case of Celecoxib, Carvedilol, Sunitinib and Allopurinol, while in case of Furosemide, Milli-Q water was used for dilution. Thereafter, they were subjected to Ultraviolet-visible (UV-Vis) NanoDrop 2000c spectrophotometer (Thermo Fisher Scientific, USA) for taking absorbance of the sample against blank micelles. The absorbances of furosemide, celecoxib, carvedilol and sunitinib were measured at λmax 333nm, 253 nm, 331nm, 425nm, respectively. Allopurinol samples were analysed using HPLC (Agilent 1100 series, C-18 column Inertsil ODS-3 5µm, 4.6 x150mm) using methanol: water 1:10 at flow rate 1ml/min and retention time for drug was found at 7 min (λ_{max} 249 nm). The drug concentration was determined by plotting the absorbance value against concentration in the standard calibration curve. All studies were conducted in triplicates in which DC wt % and EE % were calculated using the formulas below:

$$\text{DC wt\%} = \frac{\text{Weight of loaded drug}}{\text{Weight of polymer used}} * 100$$

$$\text{EE\%} = \frac{\text{Weight of loaded drug}}{\text{Weight of drug in feed}} * 100$$

Particle size and Zeta potential

ZetaSizer NanoZS® (Malvern Instruments, UK) was used to measure average particle size and polydispersity index of the micelles in which samples were diluted to 100 µg/mL with respect to polymer concentration with MilliQ water and transferred into respective cuvettes for analysis. Measurements were performed at 25 °C. Surface zeta potential was measured with the same instrument in HEPES 25 mM buffer (pH 7.2) for polymer concentration of 50 µg/ml.

Thermal properties

The thermal properties of mPEG-b-PJL and mPEG-b-PJL-COOH were analysed using DSC 250 instrument (TA instrument). The heat-cool-heat method was used under nitrogen gas with a flow of 50 mL/min. The samples were analysed between -90 to 100 ˚C. The heating and cooling rates were 10 ˚C/min and 20˚C/min respectively.

Computational Studies:

Polymer preparation

To study the interactions of the drug molecules with the studied polymers, atomic scale models of the polymers were created. To prepare a poly(jasmin lactone) copolymer, first polyethylene glycol 5000 (PEG5000) polymer was prepared, followed by preparation of the block copolymer mPEG-b-PJL. Briefly, the initiator and terminator end groups for the polymer were selected and the monomer of ethylene glycol was sketched using the Polymer Builder tool of Schrödinger's Materials Science suite release 2021-4 (Schrödinger, LLC, New York, NY, 2021). Similarly, the polymeric structure of poly(jasmin lactone) was created. This was followed by co-polymerisation where individual chains of both PEG5000 and poly(jasmin lactone) were co-polymerised to get mPEG-b-PJL polymer. To prepare mPEG-b-PJL-COOH copolymer, a similar procedure was adopted using the acid-functionalised monomers of poly(jasmin lactone).
To prepare the Soluplus® polymer, first plain polymeric structure of PEG6000, vinyl-caprolactam and vinyl acetate were prepared. The initiator and terminator end groups were selected and the

monomers of ethylene glycol, vinyl-caprolactam and vinyl acetate were sketched to get 13, 57 and 30-monomer-long polymers, respectively. The head and tail groups of the plain structures of PEG6000, vinyl-caprolactam and vinyl acetate were defined to get the Soluplus® co-polymer.
For all polymer structures, the backbone dihedral angle was set to random. The clashes between atoms pairs were avoided by specifying the van der Waals scale factor of 0.50 with a random seeding option.

Ligand preparation, pKa prediction and selection of correct ionization states
The 3D structures of the selected active pharmaceutical ingredients (APIs) were taken from the PubChem database[3] and processed using the LigPrep tool of the Maestro software suite (Schrödinger Release 2021-4: Schrödinger, LLC, New York, NY, 2021). The structures were desalted, and the possible tautomeric forms were generated at pH 7.0±2.0 with Epik[4]. The stereochemistry of each molecule was defined by the downloaded 3D structure. Finally, all the structures were energy minimized using the OPLS4 force field[5]. Using the MarvinSketch21.13 pKa prediction tool (ChemAxon Ltd.), pH distribution charts were created. The predicted pKa from Epik was used to set the minimum basic and acidic pKa at 298 K of each polymer and API molecule including all tautomers in the macro pKa mode[4].

Simulation system preparation

To prepare a simulation system for a set number of polymers, API and water molecules, the Disordered System Builder panel of the Schrödinger Materials Science suite was used. The maximum number of polymer chains in each system was set to five. Each system had an initial density of 0.5 g/cm3 and periodic boundary conditions (PBC) with an orthorhombic unit cell were used for all simulations. The initial disordered system was set to an 'amorphous system' using the OPLS4 force field[5]

Molecular Dynamics Simulations
Each polymeric system with the API molecules was submitted to a 500-ns molecular dynamics (MD) simulation. The simulations were performed using the multistage MD simulation workflow of Desmond (Schrödinger Release 2021-4: Desmond Molecular Dynamics System, D. E. Shaw Research, New York, NY, USA, 2021. Maestro-Desmond Interoperability Tools, Schrödinger, New York, NY, USA, 2021)[6], consisting of a 6-stage compressive relaxation protocol followed by a 5-ns Brownian dynamics (BD) simulation and finally the production MD simulation and analysis. Briefly, the compressive relaxation protocol involved 10 ps of Brownian dynamics (BD) at 10 K to remove steric clashes, followed by an annealing step at 300 K in the NVT ensemble and a 1-fs time step and continued to another 200-ps at 700 K. The next step of compressive relaxation performed for 25-ps MD simulation in the NPT ensemble at 300 K and 1.01325 bar with a 1-fs time step, followed by a 200-ps MD simulation with a 2-fs time step. In the final stage of compressive relaxation performed for 10-ns in the NPT ensemble at 300 K and 1013.25 bar with pressure to increase the density of simulation box. In the final stage, a 10-ns MD simulation in the NPT ensemble was completed using anisotropic coupling and a 2-fs time step. The production simulations were then performed for 500 ns at 300 K and 1.01325 bar using the Nose-Hoover chain thermostat[7], [8] and barostat using the Martyna-Tobias-Klein method[9] with isotropic coupling. The Coulombic method used for long-range interactions was U-series[10] while the cut-off radius for short-range interactions was set to 9.0 Å. Various bulk properties derived from the simulation trajectories were calculated using the Simulation Event Analysis panel of the Schrödinger Materials Science suite. The hydrogen and other non-bonding interactions were further analysed from the simulation data using Microsoft Excel360.

In vitro release study

The release profile of Sunitinib from mPEG-b-PJL and mPEG-b-PJL-COOH micelles was determined by a dialysis method[11] using PBS (pH 7.4) and acetate buffer (pH 4) as release media. Briefly, Sunitinib-loaded micelles solution was diluted with appropriate release media and then placed in dialysis tubing (Float-A-Lyzer) having the molecular weight cut off (mwco) of 3.5–5 kDa. The samples were dialysed against 800 mL of respective buffer at room temperature (24°C±0.5) under constant shaking. Samples (4 uL) were withdrawn directly from the dialysis tubing at predetermined time intervals and analysed by UV-Vis spectrophotometer at 425 nm against respective buffer as blank. The percentage of the cumulative amount of Sunitinib released was plotted as a function of time. Experiments were

repeated three times and the results were expressed as the mean value ± S.D. The release experiment was also performed at 37°C±0.5 for Sunitinib loaded mPEG-b-PJL-COOH micelles.

In vitro Cytotoxicity Studies

The human cervical carcinoma HeLa cell (ATCC) were used for in vitro studies. The cells were cultured in high-glucose Dulbecco's Modified Eagle Medium (DMEM) supplemented with 1X MEM Non-Essential Amino Acids Solution, 10% FBS, 1% penicillin–streptomycin and 2 mM L-glutamine at 37 °C, in a humidified incubator with 5% CO_2. AlamarBlue^{TM} cell viability assay was used to determine the toxicity of blank and sunitinib loaded micelles. HeLa cells were incubated overnight in a 96-well-plate ($5x10^3$ cells/ 100 µl media/ well) in cell growth media at 37 °C with 5% CO_2. The following day, the cells were treated with fresh media containing blank (0.25, 0.5, and 1.0 mg/mL), and sunitinib (1.25, 1.66, and 2.5 µg/mL equivalent to sunitinib concentration) loaded mPEG-b-PJL, mPEG-b-PJL-COOH and Pluronic micelles. After 68 h incubation at 37 °C, 5% CO_2, 10 µl of AlamarBlue cell proliferation reagent was added and the plate was incubated for further 4 h. The fluorescence of reduced AlamarBlue was then measured according to the manufacturer's protocol (Ex. 560 nm, Em. 590 nm) in Thermo Scientific VarioSkan Flash plate reader. The percentage cell proliferation was reported relative to cells treated only with cell media (100% viability).

Ex vivo haemolytic study

Blank micelles solution of mPEG-b-PJL and mPEG-b-PJL-COOH of different concentrations were used in the haemolytic study following a reported procedure with minimal modification[12]. In brief, micelles (50 mg/mL) were synthesized in PBS and were further diluted with PBS to make 25, 12.5, 1.25 and 0.625 mg/mL concentration. Human blood (5 mL) was withdrawn directly from an anonymous donor into Na_2-EDTA-coated tube to prevent coagulation. The collected sample was then centrifuged at 500 g for 5 min to separate red blood cells (RBCs) from plasma and plasma (yellowish upper layer) was discarded. 150 mM NaCl solution was used to wash RBCs twice followed by one wash with PBS (pH – 7.4). Thereafter, RBCs were diluted up to 5 times with PBS to make a stock suspension.

For the haemolysis assay, 800µL were taken from each micelle concentration and were made up to 1mL through adding 200 µL of RBCs suspension stock and hence the stocks of 50, 25, 12.5, 1.25 and 0.625 mg/mL were diluted to a final concentration of 40, 20, 10, 1 and 0.5 mg/mL of micelles, respectively. For the preparation of positive control tubes, 800 µl of 1.25% solution of triton X-100 were added to 200 µl RBCs while 800 µl of PBS was added for the preparation of negative control tubes. Tubes (n = 3) were then incubated at 37 °C for 1 h and for 24 h separately with shaking. The Tubes were then centrifuged for 5 min at 500g to pellet undamaged RBCs and supernatant from each tube was analysed using UV–Vis spectrophotometer to measure the absorbance of released haemoglobin (λ_{max} – 414nm) and the below formula was used to calculate the percentage of haemolysis:

$$\% \text{ Haemolysis} = \frac{\text{Abs of sample - Abs of negative control}}{\text{Abs of positive control - Abs of negative control}}$$

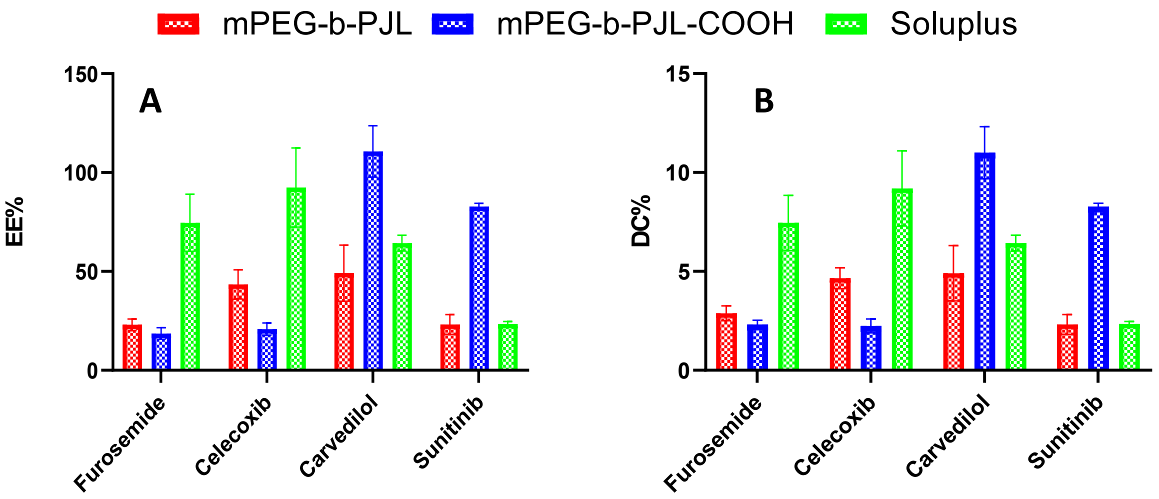

Figure S- 1 (A) Encapsulation efficiency (EE%) and (B) Drug content (DC wt%) of acidic and basic drugs under study in different block copolymeric micelles. The drug in feed for the reported data in the graph is 0.5mg/ml.

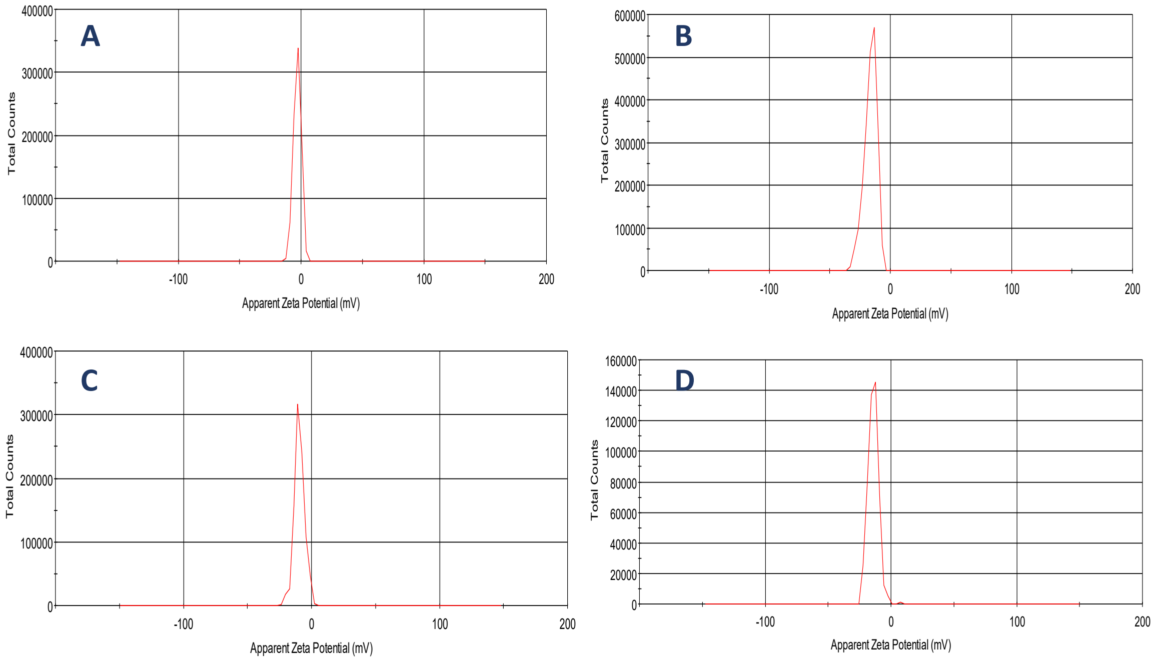

Figure S- 2 Zeta potential (mv) of blank mPEG-b-PJL (A) blank mPEG-b-PJL-COOH (B) and sunitinib loaded mPEG-b-PJL (C) mPEG-b-PJL-COOH (D) PMs at 25 °C, in 25mM Hepes buffer at pH 7.2

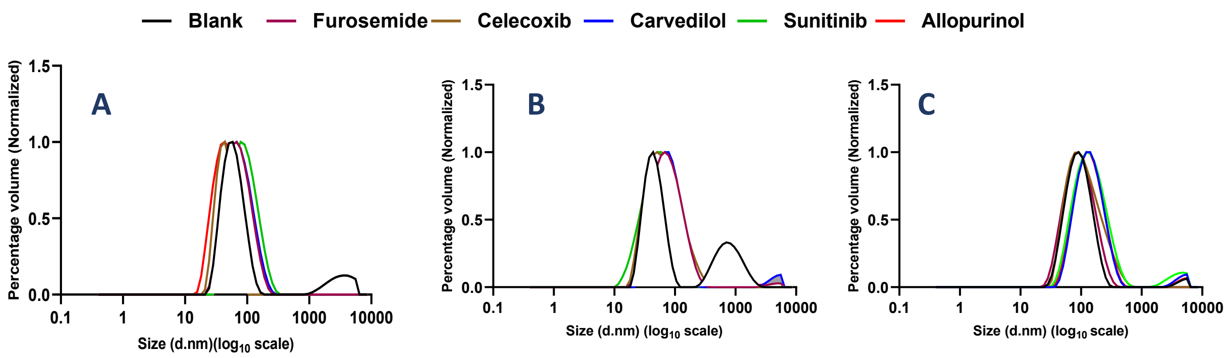

Figure S- 3 Size distribution curve by volume determined by DLS for (A) blank and drug loaded mPEG-b-PJL (B) blank and drug loaded mPEG-b-PJL-COOH and (C) blank and drug loaded Soluplus® micelles.

Table S-1 - Drug content in wt%- and encapsulation efficiency% of carvedilol and sunitinib loaded in mPEG-b-PJL, mPEG-b-PJL-COOH and Soluplus® micelles at feed ration of 0.5 mg/mL. (SD- Standard deviation).

Sample	DC wt% ± SD	EE% ± SD
Carvedilol loaded mPEG-PJL	4.9 ± 1.40	49.16 ± 14.19
Carvedilol loaded mPEG-PJL-COOH	11 ± 1.32	110.72± 13.02
Carvedilol loaded Soluplus®	6.43± 0.40	64.34± 4.01
Sunitinib loaded mPEG-PJL	2.31 ± 0.49	23.17 ± 4.96
Sunitinib loaded mPEG-PJL-COOH	8.28 ± 0.17	82.81 ± 1.68
Sunitinib loaded Soluplus®	2.34 ± 0.11	23.46± 1.19

*The data for Furosemide and Celecoxib is reported in Table 1

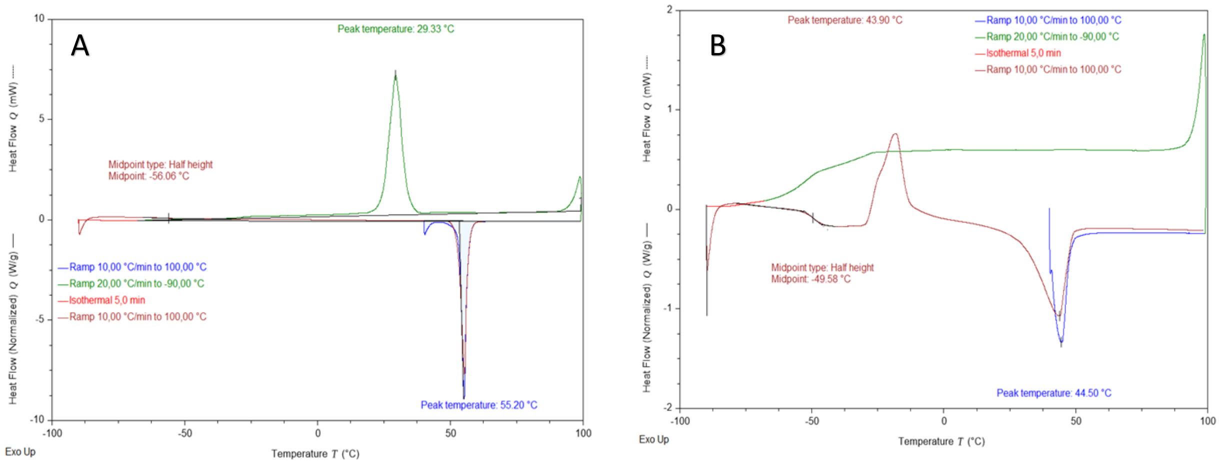

Figure S- 4 DSC analysis of mPEG-b-PJL showing glass transition (T_g) and melting temperature and (B) DSC analysis of mPEG-b-PJL-COOH showing glass transition (T_g) and melting temperature

References

[1] K. K. Bansal, E. Özliseli, A. Rosling, and J. M. Rosenholm, “Synthesis and Evaluation of Novel Functional Polymers Derived from Renewable Jasmine Lactone for Stimuli-Responsive Drug Delivery,” *Advanced Functional Materials*, vol. 31, no. 33, pp. 1–8, 2021, doi: 10.1002/adfm.202101998.

[2] M. Gou *et al.*, “Curcumin-loaded biodegradable polymeric micelles for colon cancer therapy in vitro and in vivo,” *Nanoscale*, vol. 3, no. 4, pp. 1558–1567, 2011, doi: 10.1039/c0nr00758g.

[3] S. Kim *et al.*, “PubChem in 2021: New data content and improved web interfaces,” *Nucleic Acids Research*, vol. 49, no. D1, pp. D1388–D1395, Jan. 2021, doi: 10.1093/nar/gkaa971.

[4] J. C. Shelley, A. Cholleti, L. L. Frye, J. R. Greenwood, M. R. Timlin, and M. Uchimaya, “Epik: A software program for pKa prediction and protonation state generation for drug-like molecules,” *Journal of Computer-Aided Molecular Design*, vol. 21, no. 12, pp. 681–691, Dec. 2007, doi: 10.1007/s10822-007-9133-z.

[5] C. Lu *et al.*, “OPLS4: Improving Force Field Accuracy on Challenging Regimes of Chemical Space,” *Journal of Chemical Theory and Computation*, p. acs.jctc.1c00302, Jun. 2021, doi: 10.1021/acs.jctc.1c00302.

[6] F. Sievers *et al.*, “Fast, scalable generation of high-quality protein multiple sequence alignments using Clustal Omega,” *Molecular Systems Biology*, vol. 7, no. 1, p. 539, Jan. 2011, doi: 10.1038/msb.2011.75.

[7] S. Nosé, “A molecular dynamics method for simulations in the canonical ensemble,” *Molecular Physics*, vol. 52, no. 2, pp. 255–268, 1984, doi: 10.1080/00268978400101201.

[8] S. Nosé, “A unified formulation of the constant temperature molecular dynamics methods,” *The Journal of Chemical Physics*, vol. 81, no. 1, pp. 511–519, Jul. 1984, doi: 10.1063/1.447334.

[9] G. J. Martyna, D. J. Tobias, and M. L. Klein, “Constant pressure molecular dynamics algorithms,” *The Journal of Chemical Physics*, vol. 101, no. 5, pp. 4177–4189, Sep. 1994, doi: 10.1063/1.467468.

[10] C. Predescu *et al.*, “The u -series: A separable decomposition for electrostatics computation with improved accuracy,” *Journal of Chemical Physics*, vol. 152, no. 8, p. 084113, Feb. 2020, doi: 10.1063/1.5129393.

[11] Z. Ma, A. Haddadi, O. Molavi, A. Lavasanifar, R. Lai, and J. Samuel, “Micelles of poly(ethylene oxide)-b-poly(ε-caprolactone) as vehicles for the solubilization, stabilization, and controlled delivery of curcumin,” *Journal of Biomedical Materials Research - Part A*, vol. 86, no. 2, pp. 300–310, 2008, doi: 10.1002/jbm.a.31584.

[12] B. C. Evans *et al.*, “Ex vivo red blood cell hemolysis assay for the evaluation of pH-responsive endosomolytic agents for cytosolic delivery of biomacromolecular drugs.,” *J Vis Exp*, no. 73, pp. 7–11, 2013, doi: 10.3791/50166.
